# Neutrophil extracellular traps contribute to coagulopathy after traumatic brain injury

**DOI:** 10.1172/jci.insight.141110

**Published:** 2023-03-22

**Authors:** Jiaqi Jin, Fang Wang, Jiawei Tian, Xinyi Zhao, Jiawei Dong, Nan Wang, Zhihui Liu, Hongtao Zhao, Wenqiang Li, Ge Mang, Shaoshan Hu

**Affiliations:** 1 Department of Neurosurgery, Cancer Center, Zhejiang Provincial People’s Hospital, Hangzhou Medical College, Hangzhou, Zhejiang, China.; 2 Department of Neurosurgery, The Second Affiliated Hospital of Harbin Medical University, Harbin, China.; 3 Department of Cardiology, The Second Affiliated Hospital of Harbin Medical University, Harbin, China.; 4 Department of Vascular Surgery, Jinshan Hospital of Fudan University, Shanghai, China.

**Keywords:** Cell Biology, Neuroscience, Coagulation, Endothelial cells, Neutrophils

## Abstract

Coagulopathy contributes to the majority of deaths and disabilities associated with traumatic brain injury (TBI). Whether neutrophil extracellular traps (NETs) contribute to an abnormal coagulation state in the acute phase of TBI remains unknown. Our objectives were to demonstrate the definitive role of NETs in coagulopathy in TBI. We detected NET markers in 128 TBI patients and 34 healthy individuals. Neutrophil-platelet aggregates were detected in blood samples from TBI patients and healthy individuals using flow cytometry and staining for CD41 and CD66b. Endothelial cells were incubated with isolated NETs and we detected the expression of vascular endothelial cadherin, syndecan-1, thrombomodulin, von Willebrand factor, phosphatidylserine, and tissue factor. In addition, we established a TBI mouse model to determine the potential role of NETs in TBI-associated coagulopathy. NET generation was mediated by high mobility group box 1 (HMGB1) from activated platelets and contributed to procoagulant activity in TBI. Furthermore, coculture experiments indicated that NETs damaged the endothelial barrier and caused these cells to assume a procoagulant phenotype. Moreover, the administration of DNase I before or after brain trauma markedly reduced coagulopathy and improved the survival and clinical outcome of mice with TBI.

## Graphical abstract

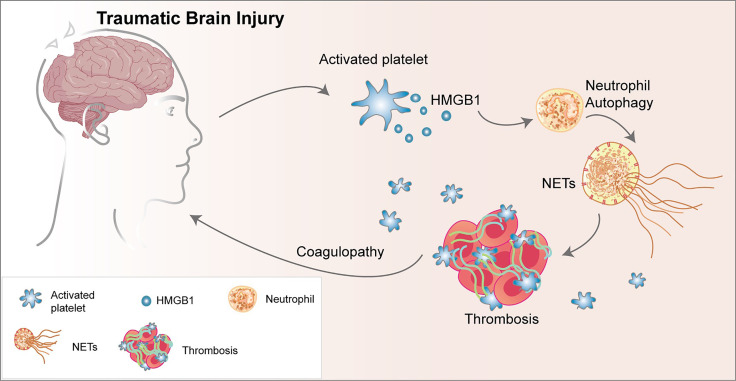

## Introduction

Traumatic brain injury (TBI), as one of the leading causes of death and disability, affects more than 50 million people each year worldwide and is increasingly recognized as an important global health problem (1, 2). Nearly two-thirds of TBI patients are diagnosed with abnormal coagulation states upon admission (3–7). Thus, there is a growing need to understand the mechanism of coagulopathy after TBI.

Despite numerous studies demonstrating poor clinical outcomes associated with coagulopathy after TBI, the underlying mechanism of coagulopathy remains poorly understood. Compared with other types of traumas, TBI induces coagulopathy in different ways due to the lack of significant hemorrhage, hypoperfusion, and shock (8, 9). In a study of 345 TBI patients, D-dimer and fibrinogen degradation products dramatically changed in a time-dependent manner (10). In another study of 242 TBI patients, disseminated intravascular coagulation was observed in TBI patients with coagulopathy (11). These studies indicate that TBI-associated coagulopathy is a complicated state consisting of dynamic changes in the coagulation and fibrinolytic systems.

A widely accepted phenomenon is that neutrophils are among the first responders to injured tissue both in the periphery and in the CNS (12, 13). Previous studies revealed that neutrophils are the most abundant circulating cells after TBI and upregulate the expression of various membrane receptors and granule proteins that are critical for the pathophysiology of TBI (14, 15). For trauma patients, the delayed apoptosis of neutrophils is an important reason why neutrophils can participate in the pathophysiology of diseases for extended periods (16). Unfortunately, relatively little is known about the fate and definitive function of neutrophils with delayed apoptosis in the acute phase of TBI. Neutrophil extracellular traps (NETs) are composed of extracellular chromatin decorated with histones, proteases, and other granule proteins (17, 18). Increasing evidence has emerged suggesting that NETs play a role in coagulation diseases, including stroke, sepsis, and atherosclerosis (19–22). Activated neutrophils were recently reported to generate NETs in traumatic brain injury and contribute to exacerbating neurological deficits in animal models (23, 24). However, the definitive role of NETs in TBI-associated coagulopathy remains largely unknown. Neutrophil-platelet aggregate (NPA) formation has been reported as a critical process in trauma-induced coagulopathy (25). It is well established that activated platelets are important mediators in NET formation in various diseases (26–28). However, the interaction between platelets and NET formation in TBI and its potential role in TBI-associated coagulopathy is poorly understood. Endothelial damage commonly occurs in trauma patients and can initiate and exacerbate coagulopathy in the acute phases of diseases (29, 30). However, how the interaction between NETs and endothelial cells (ECs) might affect the process of coagulopathy after TBI remains unknown.

In this study, we show that the delayed apoptosis of neutrophils enhanced the formation of NETs and induced coagulopathy in the acute phase of TBI. Activated platelets promote NET generation by releasing high mobility group box 1 (HMGB1) in TBI patients. Moreover, NETs initiated procoagulant activity by binding coagulation factors through the exposure of phosphatidylserine (PS) and the expression of tissue factor (TF). In addition, due to the importance of endothelial dysfunction in the coagulation state in TBI, we investigated the interaction between NETs and ECs. Our study may help to identify the potential mechanisms of TBI-associated coagulopathy and to explore novel therapeutic targets for intervention and prevention in TBI patients.

## Results

### Delayed apoptosis of neutrophils from TBI patients is associated with the formation of extracellular chromatin.

**Figure 1:**
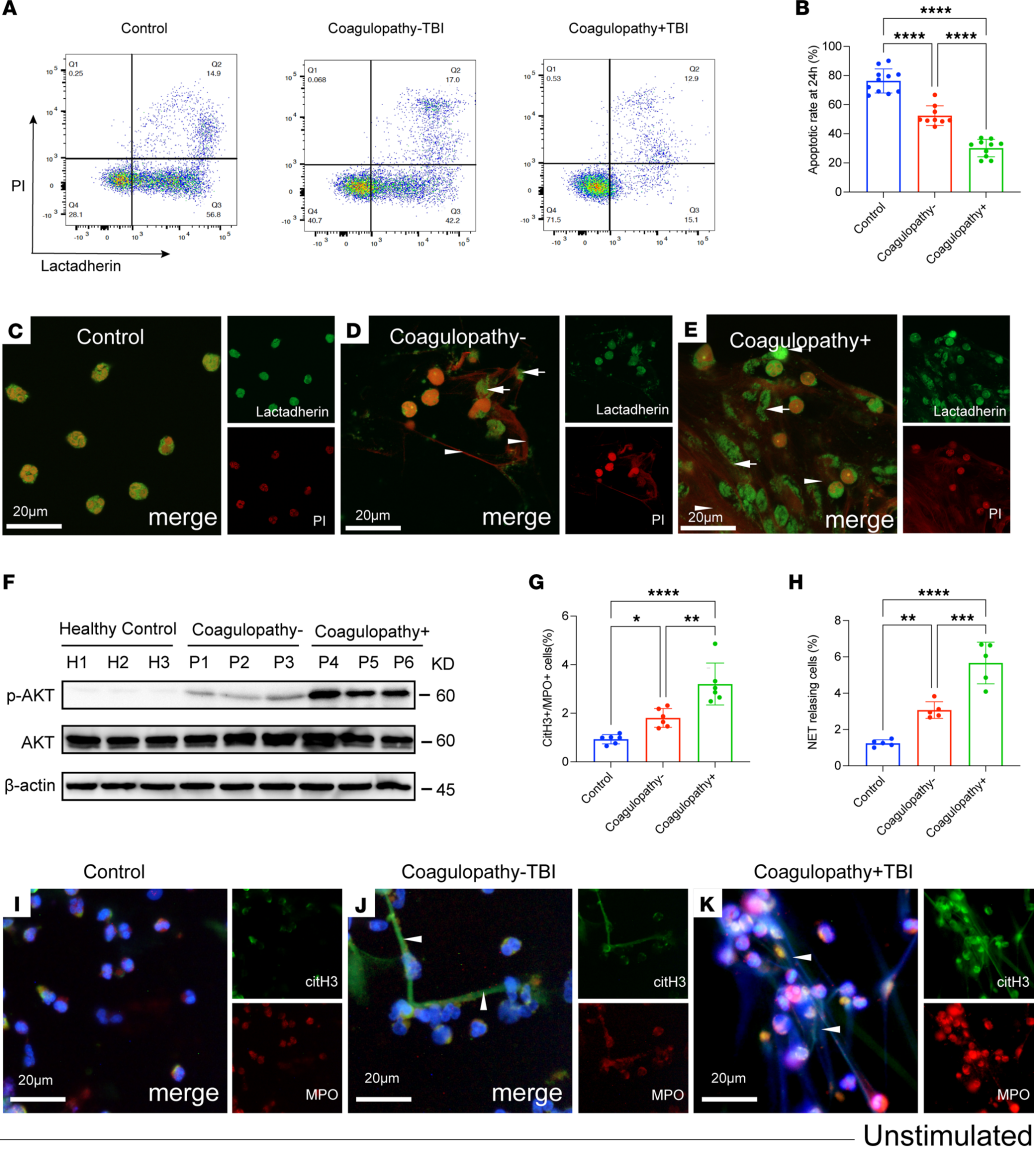
Delayed apoptosis of neutrophils from TBI patients is associated with extracellular traps. (**A**) Apoptosis of cultured neutrophils from healthy individuals (*n* = 11), noncoagulopathic patients (coagulopathy–, *n* = 9), and coagulopathic patients (coagulopathy+, *n* = 10) was measured by flow cytometry. (**B**) Percentage of apoptotic neutrophils from each group after culture for 24 hours. (**C**–**E**) Representative confocal microscopy images of cultured neutrophils from healthy controls, noncoagulopathic patients, and coagulopathic patients stained with lactadherin (green, arrows) and PI (red, arrowhead). (**F**) The expression of p-AKT and AKT on neutrophils from healthy controls (H1–H3), coagulopathy– patients (P1–P3), and coagulopathy+ patients (P4–P6) was detected by Western blotting. (**G**) NETting neutrophils, defined as MPO^+^citH3^+^, were detected in samples from each group by flow cytometry. (**H**–**K**) Neutrophils from healthy individuals and TBI patients were stained for MPO (red) and citH3 (green) and observed by confocal microscopy. Scale bars: 20 μm (**C**–**E** and **I**–**K**). Data are presented as the mean ± SD. *P < 0.05; **P < 0.01; ***P < 0.001; ****P < 0.0001 by 1-way ANOVA with Tukey’s multiple-comparison test (**B**, **G**, and **H**).

To evaluate the apoptotic state of neutrophils from healthy individuals, neutrophils from TBI patients with (coagulopathy+ TBI) and without coagulopathy (coagulopathy– TBI) were stained with propidium iodide (PI) and lactadherin, which is used as a probe for detecting PS (31), and analyzed by flow cytometry. Control neutrophils displayed more complete apoptosis at 24 hours than TBI patient neutrophils, especially those with coagulopathy (Figure 1, A and B). To compare the differences in neutrophils from healthy individuals and TBI patients, cultured neutrophils were stained with lactadherin and PI and analyzed by confocal microscopy (Figure 1, C–E). Our results showed that more NETosis than apoptosis was observed in neutrophils from TBI patients (Figure 1, C–E). Akt has been reported to be involved in redirecting NETosis to apoptosis. We also observed that the expression of phosphorylated-Akt (p-Akt) in Western blots of cultured neutrophils from TBI patients, especially those with coagulopathy, was obviously increased compared with those from healthy donors (Figure 1F) (32). Moreover, neutrophils from coagulopathic patients tended to generate more NETs than those from patients without coagulopathy, as observed by flow cytometry (Figure 1G) and confocal microscopy (Figure 1, H–K).

### Neutrophil-platelet aggregates are primed to form NETs during TBI.

**Figure 2:**
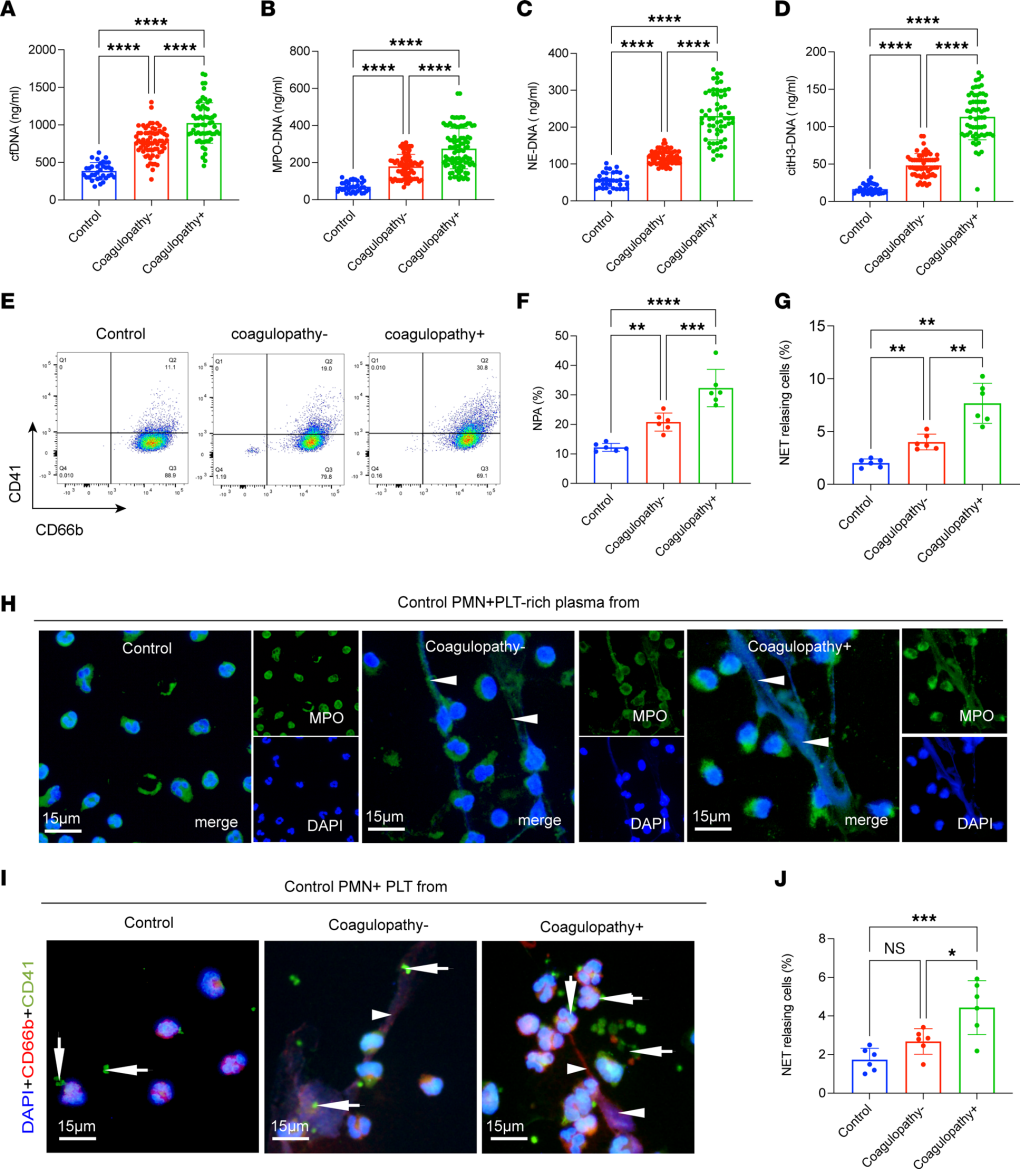
Neutrophils from TBI patients are primed to form NETs. (**A**–**D**) The circulating NET markers cfDNA, MPO-DNA, NE-DNA, and citH3-DNA were measured in plasma samples from control individuals (*n* = 34), noncoagulopathic TBI patients (*n* = 68), and coagulopathic TBI patients (*n* = 60) by ELISA. (**E**) CD66b^+^CD41^+^ events, defined as neutrophil-platelet aggregates (NPAs), were detected in samples from each group. (**F**) The proportion of NPAs in each group. Control neutrophils were incubated with PRP from each group. (**G** and **H**) The proportion of NETs was significantly elevated in cells incubated with platelet-rich (PLT-rich) plasma from coagulopathic patients. (**I** and **J**) Control neutrophils treated with platelets from each group were quantified to determine the percentage of NET-releasing cells. Scale bars: 15 μm (**H** and **I**). One representative out of 6 (**G**) independent experiments is shown. Data are presented as the mean ± SD. *P < 0.05; **P < 0.01; ***P < 0.001; ****P < 0.0001 by Brown-Forsythe and Welch’s ANOVA test (**A**, **C**, and **G**), Kruskal-Wallis test with Dunn’s multiple-comparison test (**B** and **D**), or 1-way ANOVA with Tukey’s multiple-comparison test (**F** and **J**).

To quantify NETs in TBI patients, we measured cell-free DNA (cfDNA), myeloperoxidase-DNA (MPO-DNA), neutrophil elastase–DNA (NE-DNA), and citrullinated histone H3–DNA (citH3-DNA) in plasma collected from patients with TBI and healthy individuals (Figure 2, A–D). The 4 markers of NETs, especially in those with coagulopathy, were markedly elevated in TBI patients compared with healthy donors (Figure 2, A–D). Neutrophil-platelet aggregate (NPA) formation is a critical pathophysiologic process in trauma-induced coagulopathy, as previously reported (25). Activated platelets have also been identified as important mediators of NET formation in various diseases (22, 26–28). To gain further insight into the involvement of the interactions between platelets and NETs in TBI, we detected PNAs in samples from each group. CD66b^+^CD41^+^ events, defined as NPAs, were significantly elevated in samples from coagulopathic patients (Figure 2, E and F). Then, control neutrophils were treated with platelet-rich plasma (PRP) from each group to investigate the contribution of the inflammatory environment of TBI to NET formation. PRP from TBI patients, especially those with coagulopathy, caused neutrophils to generate more NETs than control PRP, as observed by confocal microscopy (Figure 2, G and H). In addition, we incubated control neutrophils with platelets from each group. Confocal images revealed that platelets from coagulopathic patients were the strongest initiators of NET formation, suggesting the critical role of platelets in NET formation in the acute phase of TBI (Figure 2, I and J).

### HMGB1 from activated platelet–mediated neutrophil autophagy promotes NET formation.

**Figure 3:**
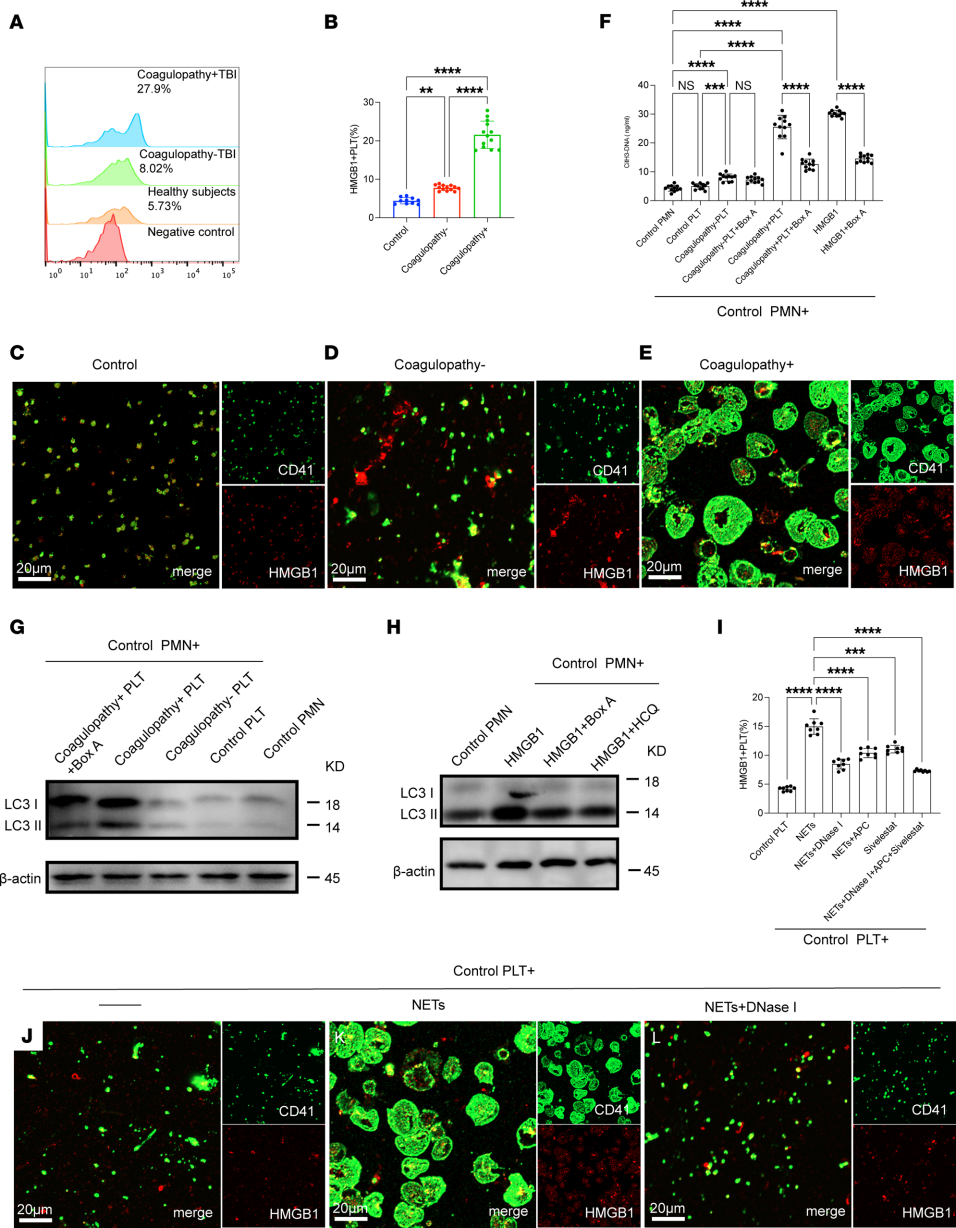
HMGB1 from platelet-mediated neutrophil autophagy promotes NET formation. (**A**) HMGB1^+^ platelets were detected in whole blood samples from healthy individuals and patients and analyzed by flow cytometry. (**B**) The proportion of HMGB1^+^ platelets was significantly elevated in samples from TBI patients, especially those with coagulopathy. (**C**–**E**) Platelets from each group stained for HMGB1 (red) and CD41 (green) and observed by confocal microscopy. (**F**) Control neutrophils were cultured with platelets (PLT) from each group in the presence of Box A (HMGB1 competitive antagonist). The concentration of citH3-DNA complexes in the culture supernatant was measured by ELISA. (**G**) The expression of LC3B on neutrophils treated with platelets from each group. (**H**) Control neutrophils were treated with HMGB1 in the presence of HCQ (autophagy inhibitor) or Box A. The expression of LC3B on neutrophils from each group was detected by Western blotting. (**I**) Control platelets were incubated with NETs (0.5 μg DNA/mL) or with NETs in the presence of NET inhibitors (DNase I, APC, sivelestat). HMGB1^+^ platelets were detected by flow cytometry. (**J**–**L**) Representative images of platelets incubated with NETs (0.5 μg DNA/mL) or with NETs plus DNase I. Scale bars: 20 μm (**C**–**E** and **J**–**L**). Data are presented as the mean ± SD. **P < 0.01; ***P < 0.001; ****P < 0.0001 by 1-way ANOVA with Tukey’s multiple-comparison test (**B** and **J**) or Brown-Forsythe and Welch’s ANOVA test (**F** and **I**).

Next, we investigated the interaction between platelet activation and NET formation in TBI. We first detected platelet activation by flow cytometry. Our results showed that platelets from TBI patients, especially those with coagulopathy, were more likely to express HMGB1 than platelets from other groups (Figure 3, A and B). Confocal images also showed a similar trend to that of flow cytometry (Figure 3, C–E). Control neutrophils were incubated with platelets from each group. The concentration of citH3-DNA complexes in the culture supernatant suggested that HMGB1 from platelets induces NET formation in TBI (Figure 3F). HMGB1 has been described as a potent initiator of autophagy (22, 27). Western blots showed the expression of LC3B on neutrophils treated with platelets from TBI patients, especially those with coagulopathy, and these effects could be decreased with Box A (HMGB1 competitive antagonist) (Figure 3G). In addition, hydroxychloroquine (HCQ) and Box A could both attenuate neutrophil autophagy (Figure 3H). Moreover, we observed that the expression of PS and TF on DNA traps was dependent on HMGB1 concentration (Supplemental Figure 2, A–F; supplemental material available online with this article; https://doi.org/10.1172/jci.insight.141110DS1). Based on our flow cytometry findings of high levels of NPAs in TBI patients, we examined the potential role of NETs in platelet activation in TBI. The expression level of platelet HMGB1 was significantly elevated when incubated with isolated NETs by flow cytometry (Figure 3, A and B) and confocal microscopy (Figure 3, C–E). Moreover, Western blotting results showed that HMGB1 on platelets treated with NETs was mainly from platelets and not just NETs (Supplemental Figure 3). However, these effects were reversed by NET inhibitors, including DNase I, activated protein C (APC), and sivelestat (Figure 3, I–L).

### NETs induce procoagulant activity in TBI.

**Figure 4:**
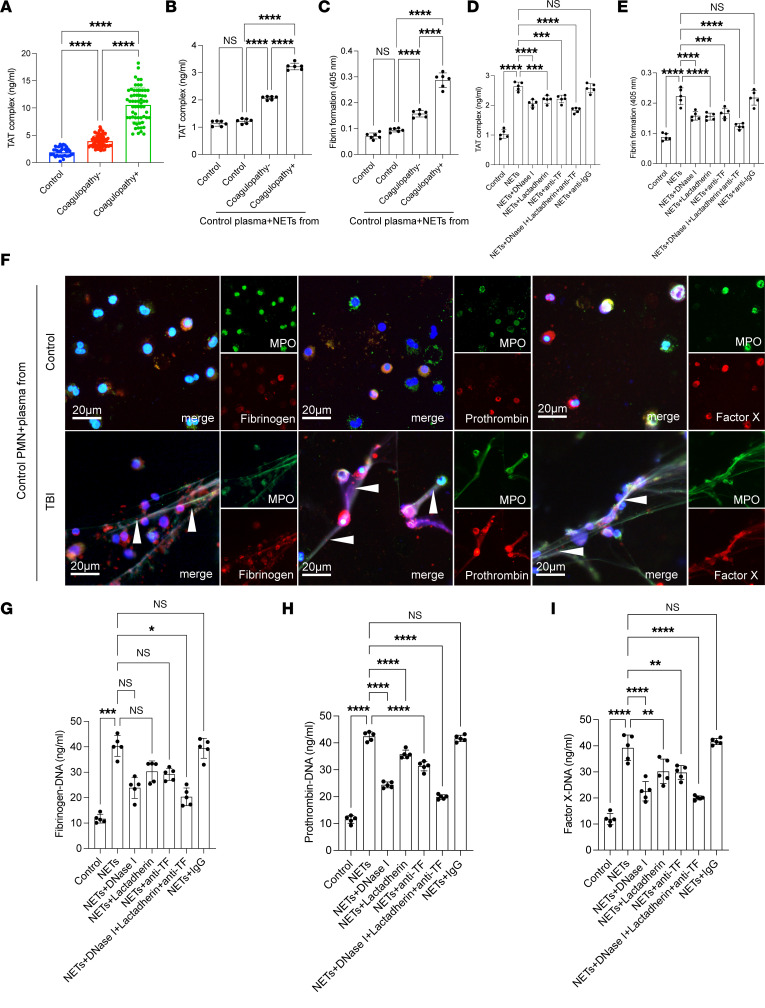
NETs promote procoagulant activity in TBI. (**A**) The TAT complex of plasma samples from healthy individuals and TBI patients was detected by ELISA. (**B**) Thrombin generation (TAT-complex) and fibrin formation (**C**) of NETs from each group was detected by ELISA. In inhibition assays, lactadherin and anti-TF antibody decreased the TAT complex (**C**) and fibrin formation (**D**) of NETs. In inhibition assays, lactadherin and anti-TF antibody decreased the TAT complex (**D**) and fibrin formation (**E**) of NETs. (**F**) Control neutrophils were incubated with plasma from healthy controls, and TBI patients stained for coagulation factors such as fibrinogen (red), prothrombin (red), factor X (red), and MPO (green) and analyzed by confocal microscopy. Scale bars: 20 μm. Fibrinogen-DNA (**G**), prothrombin-DNA (**H**), and factor X–DNA (**I**) in each group were detected by ELISA. Data are presented as the mean ± SD. *P < 0.05; **P < 0.01; ***P < 0.001; ****P < 0.0001 by Kruskal-Wallis test with Dunn’s multiple-comparison test (**A** and **G**) or 1-way ANOVA with Tukey’s multiple-comparison test (**B**–**E**, **H**, and **I**).

There was a high level of TF and exposed PS on NETs, indicating the potential role of NETs in coagulation. We then investigated the potential mechanism for procoagulant activity of NETs in the process of TBI. We detected the thrombin generation of plasma samples from healthy individuals and TBI patients by ELISA (Figure 4A). Thrombin–anti-thrombin (TAT) complexes were significantly increased in samples from coagulopathic patients and positively correlated with the NET markers (citH3-DNA, *r* = 0.7287, *P* < 0.0001; MPO-DNA, *r* = 0.6308, *P* < 0.0001; NE-DNA, *r* = 0.6894, *P* < 0.0001; Supplemental Figure 4, A–C). We also observed that NETs from coagulopathic patients showed strong potential to generate thrombin by detecting TAT complexes (Figure 4B). In inhibition assays, in the presence of lactadherin and anti-TF antibodies, which could inhibit PS and TF, respectively, thrombin and fibrin formation in NETs was significantly diminished, suggesting that PS and TF are important mediators of the coagulant activity of NETs (Figure 4, C–E). Confocal microscopy images also showed that coagulation factors such as fibrinogen, prothrombin, and factor X were bound to NETs when neutrophils were incubated with plasma from TBI patients (Figure 4F). We also observed a similar phenomenon when control plasma was incubated with isolated NETs (Supplemental Figure 5, A–C). In contrast, few coagulation factors localized to neutrophils incubated with the control plasma (Figure 4F). In inhibition assays, the coagulation factors binding to NETs were markedly mitigated in the presence of lactadherin and anti-TF antibody (Figure 4, G–I). We detected fibrinogen-DNA, prothrombin-DNA, and factor X–DNA in each group, and we observed a similar trend when NETs were incubated with lactadherin and anti-TF antibody (Figure 4, F–H). Moreover, coagulation protein generation in platelets was dependent on NET concentration, and these effects were significantly reduced by NET inhibitors (Supplemental Figure 6, A–D).

### NETs exacerbate endothelial dysfunction in TBI.

**Figure 5:**
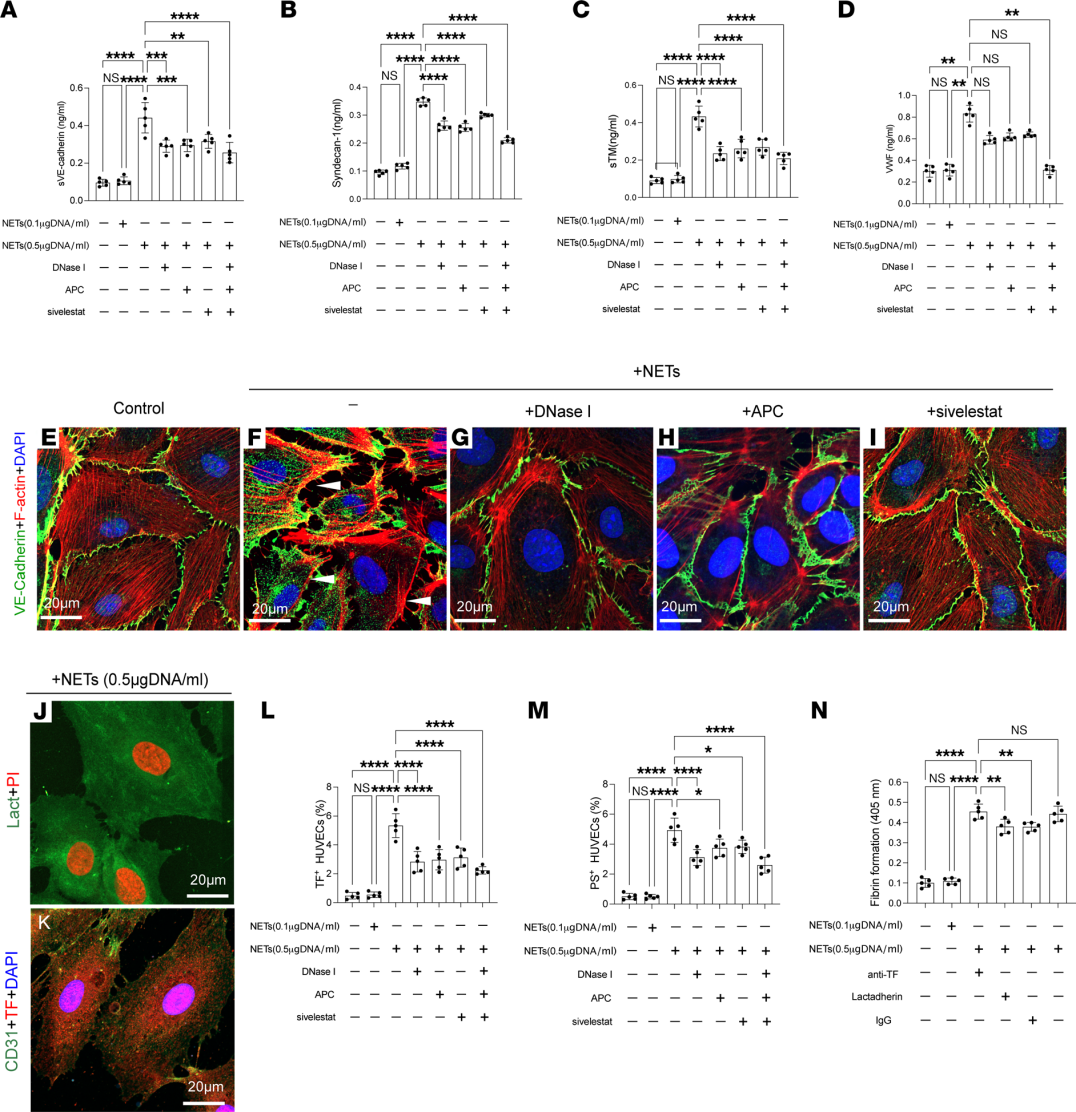
NETs promote endothelial dysfunction in TBI. ECs were incubated with isolated NETs. ELISA results suggested that soluble VE-cadherin (sVE-cadherin) (**A**), syndecan-1 (**B**), soluble thrombomodulin (sTM) (**C**), and VWF (**D**) levels in the supernatant of ECs were dependent on NET concentration. (**E**–**I**) The expression of VE-cadherin on treated ECs, analyzed by confocal microscopy. Representative confocal images of PS (**J**) and TF (**K**) on ECs incubated with NETs (0.5 μg DNA/mL). The proportion of TF (**L**) and PS (**M**) on human umbilical vein ECs (HUVECs) incubated with NETs (0.1 or 0.5 μg DNA/mL) in the presence of NET inhibitors (DNase I, APC, sivelestat). (**N**) Fibrin formation of ECs with NETs (0.1 or 0.5 μg DNA/mL) ) or in the presence of anti-TF antibody and lactadherin. Scale bars: 20 μm (**E** and **F**). Data are presented as the mean ± SD. *P < 0.05; **P < 0.01; ***P < 0.001; ****P < 0.0001 by 1-way ANOVA with Tukey’s multiple-comparison test (**A**–**C** and **L**–**N**) or Kruskal-Wallis test with Dunn’s multiple-comparison test (**D**).

EC activation is a crucial mechanism in coagulopathy after TBI. Thus, we investigated the potential relationship between NETs and EC activation. The endothelial markers syndecan-1, soluble thrombomodulin, and von Willebrand factor (VWF) were detected in plasma from each group (Supplemental Figure 7, A–C). These markers were significantly increased in plasma from coagulopathic patients compared with other groups (Supplemental Figure 7, A–C). Moreover, these markers were positively correlated with citH3-DNA in samples from coagulopathic patients (syndecan-1, *r* = 0.5848, *P* < 0.0001; soluble thrombomodulin, *r* = 0.7092, *P* < 0.0001; VWF, *r* = 0.7746, *P* < 0.0001; Supplemental Figure 7, D–F). To further substantiate the association between NETs and EC activation in TBI, we treated ECs with NETs from healthy individuals and TBI patients. ELISA results showed NETs from TBI patients, especially those with coagulopathy, could significantly impair endothelial dysfunction by decreasing the expression of syndecan-1, thrombomodulin, and VWF (Supplemental Figure 8, A–C). Moreover, we treated ECs with isolated NETs at different concentrations. ELISA results indicated that soluble vascular endothelial cadherin (VE-cadherin), syndecan-1, soluble thrombomodulin, and VWF levels in the supernatant of ECs were dependent on NET concentration. In inhibition assays, the NET inhibitors DNase I, APC (histone inhibitor), and sivelestat (NE inhibitor) significantly decreased the soluble VE-cadherin, syndecan-1, and soluble thrombomodulin levels in the supernatant of treated ECs (Figure 5, A–D). Confocal images also validated that NETs induced the destruction of the endothelial barrier based on staining for VE-cadherin (Figure 5, E–I). Furthermore, we evaluated the expression of PS and TF on ECs treated with NETs to examine thrombogenicity. The expression of PS and TF was significantly increased in ECs after treatment with NETs, and these effects were decreased by the NET inhibitors DNase I, APC, and sivelestat (Figure 5, J–M). ECs treated with NETs showed procoagulant activity, as determined by detecting fibrin formation (Figure 5N). Inhibition assays using lactadherin and anti-TF antibody suggested that ECs treated with NETs promoted coagulation activity through the expression of TF and PS exposure (Figure 5N).

### NETs induce TBI-induced coagulopathy in mouse model.

**Figure 6:**
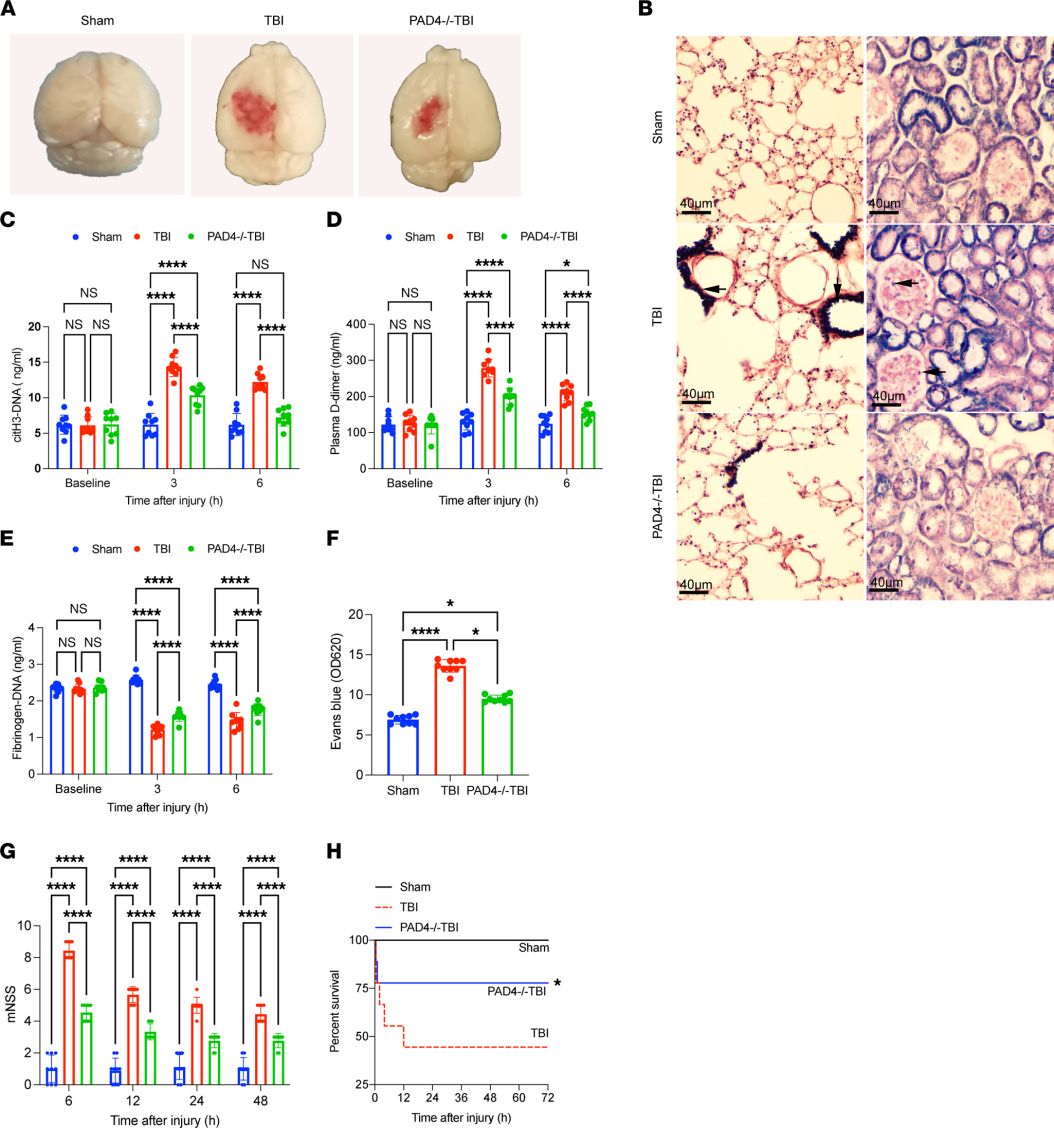
NETs induce coagulopathy in a TBI mouse model. To evaluate the potential role of NETs in coagulopathy, we established a TBI mouse model. (**A**) Representative topical views of brains from each group (sham, TBI, and PAD4^–/–^ TBI). (**B**) PTAH staining of the lungs and kidneys from each group. Fibrin deposition (black, arrow) in the microvasculature of the lungs and kidney was observed in TBI mice but not in the same organs of PAD4^–/–^ mice. Scale bars: 40 μm. Plasma levels of citH3-DNA (**C**), fibrinogen (**D**), and D-dimer (**E**) in sham (*n* = 9), TBI (*n* = 9), and PAD4^–/–^ TBI mice (*n* = 9). (**F**) Levels (OD unit) of Evans blue in the supernatants of tissue homogenates from mice in each group (*n* = 9). (**G**) Modified Neurological Severity Score (mNSS) (*n* = 9) of sham, TBI, and PAD4^–/–^ TBI mice. (**H**) Kaplan-Meier survival analysis of sham, TBI, and PAD4^–/–^ TBI mice. *P < 0.05 versus TBI mice. Data are presented as the mean ± SD. *P < 0.05; ****P < 0.0001 by 2-way ANOVA with Tukey’s multiple-comparison test (**C**–**E** and **G**) or Kruskal-Wallis test with Dunn’s multiple-comparisons test (**F**).

To evaluate the potential of NETs as a therapeutic target, we established a TBI mouse model. We observed that mice deficient in peptidylarginine deiminase 4 (PAD4^–/–^ mice) showed markedly reduced cerebral edema after trauma compared with the other groups (Figure 6A). Confocal images showed that NETs were present in brain tissue from TBI mice (Supplemental Figure 8). Phosphotungstic acid hematoxylin (PTAH) staining validated widespread fibrin deposition in the microvasculature of the lungs and kidney of TBI mice but not in the same organs of PAD4^–/–^ TBI mice (Figure 6B) (33, 34). We also detected coagulation changes after TBI in both PAD4^–/–^ and normal mice. Interestingly, PAD4^–/–^ mice showed lower levels of citH3-DNA, which was used to measure NET generation, lower plasma D-dimer levels, and higher fibrinogen levels than normal mice after TBI (Figure 6, C–E). Our results also validated that PAD4^–/–^ mice exhibited less cerebral leakage (Figure 6F) (33, 34). Moreover, PAD4^–/–^ TBI mice developed less severe neurological dysfunction, measured by Neurological Severity Score (Figure 6G) and showed reduced 3-day mortality, from 44.44% to 22.22% (Figure 6H).

### DNase I is a therapeutic target for TBI-induced coagulopathy.

**Figure 7:**
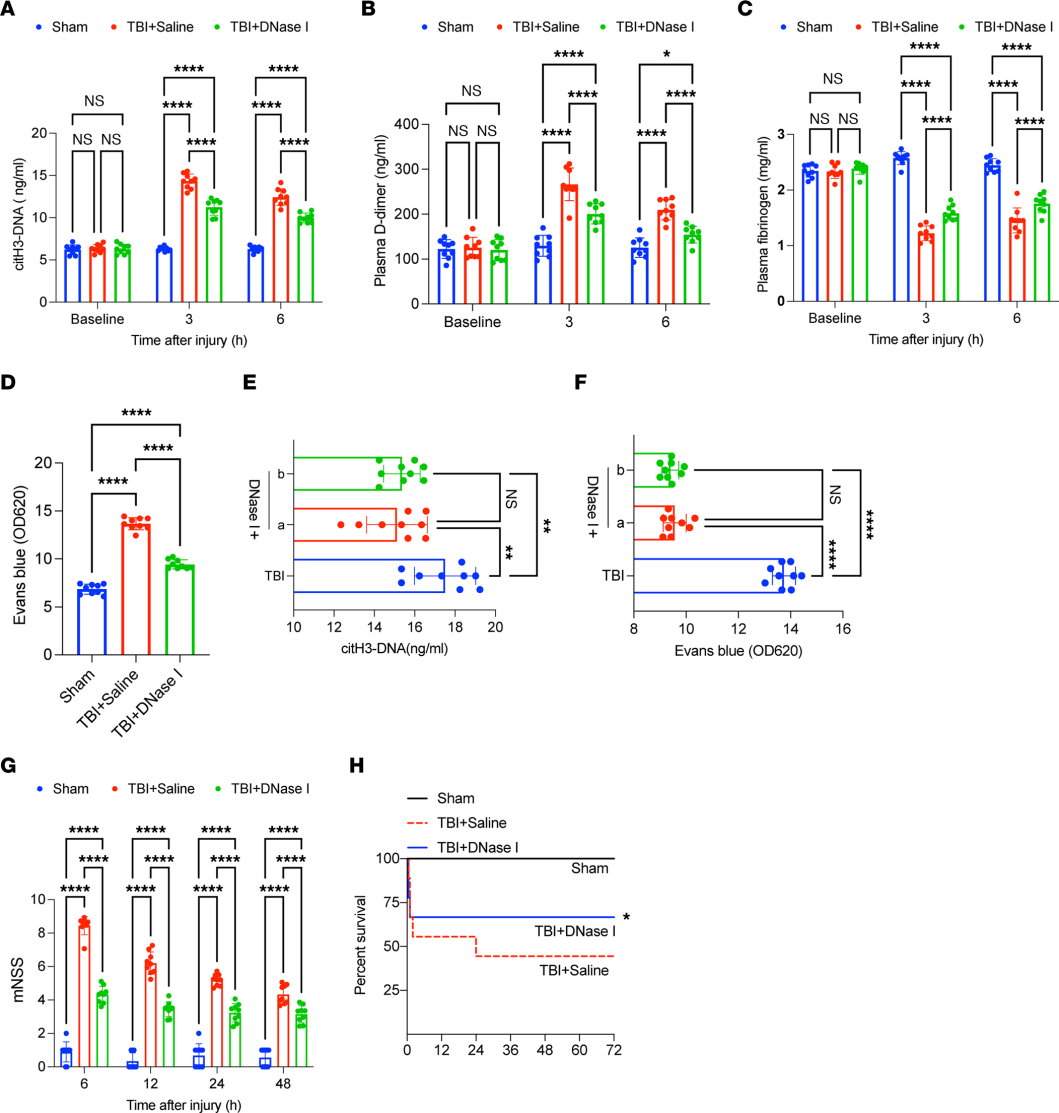
DNase I as therapeutic in TBI-associated coagulopathy. To evaluate the potential of DNase I as a therapeutic target, we established a TBI mouse model. Plasma levels of citH3-DNA (**A**), fibrinogen (**B**), and D-dimer (**C**) in sham (*n* = 9), TBI + saline (*n* = 9), and TBI + DNase I (*n* = 9) mice. (**D**) Levels (OD unit) of Evans blue in the supernatants of tissue homogenates from mice in each group (*n* = 9). Plasma levels of citH3-DNA (**E**) and levels (OD unit) of Evans blue (**F**) between mice preconditioned with DNase I (a) and those that received DNase I after injury (b). (**G**) Modified Neurological Severity Score (mNSS) (*n* = 9) of sham, TBI + saline, and TBI + DNase I mice. (**H**) Kaplan-Meier survival analysis of sham, TBI + saline, and TBI + DNase I mice. *P < 0.05 versus TBI + saline mice. Data are presented as the mean ± SD. *P < 0.05; **P < 0.01; ****P < 0.0001 by 2-way ANOVA with Tukey’s multiple-comparison test (**A**–**C** and **G**) or 1-way ANOVA with Tukey’s multiple-comparison test (**D**–**F**).

Next, we tested the effects of treatment with DNase I in a TBI mouse model. Our findings validated that DNase I significantly reduced the plasma levels of citH3-DNA and ameliorated coagulopathy, as determined by detecting D-dimer and fibrinogen (Figure 7, A–D). PTAH staining indicated DNase I could decrease fibrin deposition in the microvasculature of the lungs and kidney of TBI mice (Supplemental Figure 8, A and B). DNase I also ameliorated cerebral leakage and there were no significant differences between TBI mice treated with DNase I before and after trauma (Figure 7, E and F). TBI mice treated with DNase I also developed less severe neurological dysfunction and showed reduced 3-day mortality, from 55.56% to 33.33% (Figure 7, G and H).

## Discussion

This study reports 5 major findings. First, neutrophils from TBI patients, especially from patients who have coagulopathy, have longer survival rates, which is associated with the formation of NETs. Second, platelets from TBI patients activate neutrophils to expel NETs through autophagy mediated by HMGB1. Third, the abnormal generation of NETs bound to coagulation factors on extracellular structures contributed to procoagulant activity in TBI. Fourth, NETs induced endothelial dysfunction, including damage to the endothelial barrier and the procoagulant activity of ECs. Fifth, inhibiting NETs improved coagulopathy and clinical outcomes in a TBI mouse model.

Neutrophils are considered short-lived cells, with a half-life of approximately 1.5 and 8 hours in the circulation in mice and humans, respectively (12). In our study, an interesting finding was that neutrophils from TBI patients survived longer than neutrophils from healthy individuals due to delayed apoptosis, which was associated with the increased formation of NETs. We demonstrated that NETosis (the process of NET generation) occurred in TBI by detecting NET markers in plasma from patients and NETting neutrophils from TBI patients. Previous studies reported that delayed apoptosis of neutrophils occurs in various diseases, such as bronchiectasis, cystic fibrosis, and unstable angina (35–37). Our previous study demonstrated that cultured neutrophils obtain a procoagulant phenotype by exposing PS in vitro during a time-dependent dynamic process in apoptosis (38). In this study, we found that cultured neutrophils from TBI patients were more likely to form NETs with PS exposure than those undergoing apoptosis, indicating that NETosis is the predominant cell death mechanism in neutrophils in TBI and that NETs might have the potential to promote coagulation. Although leukocyte-platelet aggregation is tightly associated with coagulopathy in TBI, the underlying mechanism was not determined. Going beyond previous studies, we found that activated platelets promote NET formation by releasing HMGB1. Our results also showed that inhibiting HMGB1 could decrease the formation of NETs to some degree, indicating that along with platelets, inflammatory environments may also partly contribute to the generation of NETs. Further studies are needed to explore the more comprehensive mechanism of NETosis initiation in TBI.

Inflammation is a critical mechanism of secondary injury following TBI and can have complicated effects on the clinical outcome of TBI patients (39). Neutrophils, which are recognized as an important cell type in infectious diseases, mediate the connection between inflammation and coagulation by forming NETs (40). NETs have been widely evaluated in various hypercoagulable diseases, such as deep venous thrombosis (41, 42), stroke (21), and sepsis (43). However, the definitive mechanism by which NETs contribute to thrombus initiation and propagation during diseases is complicated and controversial. Noubouossie et al. reported that human neutrophil DNA, rather than intact NETs, directly promoted coagulation activation in plasma in vitro (44). Importantly, our confocal microscopy results suggested that neutrophils treated with patient plasma formed extracellular chromatin structures that bound coagulation factors, such as fibrinogen, factor Xa, and prothrombin. However, neutrophils treated with control plasma formed little extracellular chromatin and bound fewer coagulation factors, as shown in our results. Importantly, we showed that neutrophils from TBI patients formed NETs while externalizing PS, which can bind factor V and VIII to initiate the coagulation cascade, not only on the cell membrane but also on decorated extracellular structures. TF, the initiator of the extrinsic pathway, was reportedly expressed during the process of NETosis (45, 46). Treatment of isolated NETs with lactadherin and an anti-TF antibody to block PS and TF, respectively, showed that the procoagulant activity of NETs decreased significantly, suggesting that PS and TF are critical mediators of the interactions between coagulation factors and NETs.

Endothelial injury causes the activation of coagulation factors and initiates thrombin generation, which contributes to coagulopathy after TBI. In our study, ELISA results validated that syndecan-1, soluble thrombomodulin, and VWF were positively correlated with NET markers, which indicated that NETs may participate in endothelial dysfunction in TBI. Moreover, coculture experiments suggested that NETs damage the endothelial barrier by triggering PS exposure and TF expression on ECs, causing these cells to assume a procoagulant phenotype that can provide a surface that can bind coagulation factors, such as platelets, leukocytes, and fibrin. Recently, Eduardo et al. also reported that NETs induce a procoagulant phenotype in ECs through IL-1α and cathepsin G (47). Our observations added to those findings by showing that TF and PS are essential mediators of the procoagulant activity of ECs treated with NETs. ECs are critical to coagulopathy in TBI, owing to not only the direct effects on coagulation but also their role in preventing the release of procoagulant factors into the circulation, such as brain-derived microparticles (33, 34). By combining our findings and those of Allen et al. in acute brain injury, a possible mechanism may include the migration of neutrophils across the cerebrovascular endothelium and the release of NETs that contribute to neuronal death to initiate the release of brain-derived microparticles and DAMPs, which could result in uncontrolled coagulopathy in TBI (48).

Based on our encouraging in vitro results, we further validated the efficacy of inhibiting NETs in a mouse model of TBI. Using a PAD4-deficient mouse, which is not able to generate NETs, we demonstrated that blocking the generation of NETs could reduce coagulopathy after TBI. DNase I, as an effective inhibitor of NETs in various reports, could also reduce coagulopathy and improve the neurological function and survival of TBI mice. Similar results were obtained by treating with DNase I before or after TBI, suggesting that DNase I may be a novel therapeutic for coagulopathy in TBI.

Currently, although coagulopathy frequently occurs in TBI patients and has been reported elsewhere, the clinical standard of coagulopathy has not been well established (4–8, 10). According to previous studies and the coagulation standards of local hospitals, coagulopathy in our study was defined as an activated partial thromboplastin time (APTT) of greater than 42 seconds and/or an international normalized ratio (INR) of greater than 1.2 and a platelet count of less than 100 × 10^9^/L. Based on our encouraging findings in both TBI patients and in a TBI mouse model, we explored the role of neutrophils in the coagulation state in the acute phase of TBI, which is not limited to inflammatory cells. Furthermore, the observation that NETs and ECs interact may offer a possible mechanism of secondary injury in TBI, which includes an abnormal coagulation state and neurological impairment. Further investigations that detect NETs and evaluate NETs as essential markers of coagulopathy with more data are needed.

There are some limitations to our present study. First, our data were based on a relatively small size sample from a local hospital. Therefore, future studies with larger sample sizes should be conducted to confirm our conclusions. Second, our present study showed that HMGB1 from activated platelets is one of the contributing factors to NET generation in the acute phase of TBI. However, dying cells such as neurons could secrete a substantial amount of HMGB1 into the circulation. Our future work would concentrate on the in-depth mechanism underlying HMGB1 and NETs in TBI.

In summary, the delayed apoptosis of neutrophils enhanced the generation of NETs during the acute phase of TBI. This extracellular chromatin promotes coagulation through the binding of coagulation factors by PS and TF and contributes to a hypercoagulable state. NETs can also destroy the endothelial barrier and cause ECs to assume a procoagulant phenotype. Our study may also provide new insights into the abnormal coagulation changes in TBI and lead us to believe that NETs could be a novel therapeutic target for TBI-associated coagulopathy.

## Methods

### Patients.

In this study, we enrolled 34 healthy individuals and 128 TBI patients who were admitted to the Second Affiliated Hospital of Harbin Medical University from March 2017 to December 2020. Isolated TBI was defined as an intracranial abbreviated injury score (AIS) of greater than 3 and an extracranial AIS of less than 3, as previously described (10). Coagulopathy was defined as an APTT of greater than 42 seconds and/or an INR of greater than 1.2. The exclusion criteria were that the first blood sample was collected more than 1 hour after the injury; the patient had liver or renal failure, hematological disease, infections, malignancy, or was pregnant; and anticoagulants or antiplatelet agents were administered before or upon arrival at the hospital. The main characteristics of the patients and healthy controls are shown in Table 1.

### Platelets and PRP preparation.

Blood samples were collected and placed into a 5 mL tube containing 3.2% citrate. Platelet isolation was performed as follows (28): blood samples were centrifuged for 17 minutes at 150*g* at room temperature to obtain PRP. Then, 75% of the top layer of PRP was diluted with platelet wash buffer (Tianjin Haoyang Biological Manufacture, Ltd) and centrifuged at 460*g* for 17 minutes at room temperature. The isolated platelets were resuspended in prewarmed HEPES-modified Tyrode’s buffer (Solarbio).

### Platelet‑neutrophil coculture assay.

A platelet-neutrophil coculture system was established to investigate the potential role of platelets in NET generation, as previously described (26). Isolated neutrophils were seeded onto 96-well plates at 1 × 10^5^ cells per well and incubated with PRP or platelets from each group for 2 hours. The formation of NETs was quantified by ELISA (citH3-DNA). Recombinant HMGB1 protein (Sino Biological) and Box A (HMGBiotech) were used to evaluate whether HMGB1 induced NET formation in TBI (27). The formation of thrombin, extrinsic factor Xa, and intrinsic factor Xa is presented in detail in the Supplemental Methods.

### NET stimulation, isolation, and quantification.

NET stimulation, isolation, and quantification assays are described in detail in the Supplemental Methods.

### EC stimulation assays.

The human brain microvascular EC line (hcMEC/D3) was purchased from Yuchunbio. ECs were incubated for 2 hours with isolated NETs (0.1 or 0.5 μg DNA/mL) in the presence of DNase I (100 U/mL; New England Biolabs), APC (100 nM; Med Chem Express), and sivelestat (100 nM; Med Chem Express). When we cocultured ECs with NETs from healthy individuals or patients, NETs were isolated from neutrophils (5 × 10^6^ neutrophils) from healthy individuals or patients. Fibrin formation in ECs was assessed as previously described (47).

### Animal studies.

C57BL/6J mice (12–16 weeks old and 22–25 g) were purchased from the animal laboratory center of the Second Affiliated Hospital of Harbin Medical University. PAD4*^–/–^* mice on the C57BL/6J background were purchased from Cyagen Biosciences. A closed-head injury was induced using a weight-drop device, as previously described (49, 50). The experimental animals were divided into 5 groups: sham group (without brain trauma); TBI models in normal C57BL/6J background; TBI models in PAD4*^–/–^* C57BL/6J background; TBI models with DNase I (diluted in saline; New England Biolabs; 5 mg/kg) (DNase I was administered through the tail vein 1 hour before and after trauma); and TBI models with vehicle (corresponding dose of saline). PTAH staining was performed using a PTAH staining kit according to the manufacturer’s protocol (Solarbio).

### Immunofluorescence imaging.

For NET staining, isolated NETs were fixed and stained with DAPI (4′,6-diamidino-2-phenylindole) and anti-MPO (Abcam, ab25989; 1 μg/mL) and anti–histone H3 (citrulline R2+R8+R17, Abcam, ab5103; 1 μg/mL) antibodies. Percentage of NETs was quantified from 5 nonoverlapping fields per well for each sample. To visualize coagulation factor binding with NETs, treated neutrophils were stained with anti-prothrombin (Abcam, ab208589; 1 μg/mL), anti–factor Xa (Abcam, ab79929; 1 μg/mL), anti-fibrinogen (Abcam, ab34269; 1 μg/mL), and anti-MPO (Abcam, ab25989; 1 μg/mL) antibodies. ECs cultured on fibronectin-coated (5 μg/mL; Corning) slide flasks were stained with anti–VE-cadherin (Abcam, ab33168), anti–syndecan-1 (Abcam, ab33168; 1 μg/mL), anti-thrombomodulin (Abcam, ab33168; 1 μg/mL), anti-TF (Abcam, ab48647; 1 μg/mL), anti-CD31 (Abcam, ab9498; 1:500), FITC-conjugated lactadherin (Hematologic Technologies; 1 μg/mL), and TRITC-conjugated phalloidin (Yeasen; 200 nM). Platelets were stained with anti-CD41 antibody (Abcam, ab134131; 1 μg/mL), anti-HMGB1 antibody (Proteintech, 66525-1-Ig; 1 μg/mL), and anti-fibrinogen (Abcam, ab34269; 1 μg/mL). The secondary antibodies were goat anti-rabbit conjugated to Alexa Fluor 488 (Abcam, ab15077; 1 μg/mL), goat anti-rabbit conjugated to Alexa Fluor 555 (Abcam, ab15078; 1 μg/mL); goat anti-mouse conjugated to Alexa Fluor 647 (Abcam, ab150115; 1 μg/mL); and goat anti-mouse conjugated to Alexa Fluor 488 (Abcam, ab150133; 1 μg/mL). All samples were analyzed by confocal microscopy (Zeiss, LSM 800).

### Flow cytometry.

Approximately 100 μL of citrated blood freshly collected from healthy individuals and TBI patients was diluted in PBS and stained with anti-CD15 (PE, Biolegend, 301906), anti-CD16 (APC-Cy7, BD, 557758), anti-citH3 (Abcam, ab5103; 1 μg/mL), and anti-MPO (Abcam, ab25989; 1 μg/mL) antibodies, followed by a goat anti-rabbit secondary antibody conjugated to Alexa Fluor 488 (Abcam, ab15077; 1 μg/mL) and a goat anti-mouse secondary conjugated to Alexa Fluor 647 (Abcam, ab150115; 1 μg/mL). To detect NPAs, neutrophils from each group were stained with anti-CD66b (APC, Biolegend, 396906) and anti-CD41 (FITC, Biolegend, 303704). To detect activated platelets, 100 μL of fresh citrated blood or treated platelets were stained with anti-CD41 (PerCp-cy5.5, Biolegend, 303720) and anti-HMGB1 (PE, Biolegend, 651404) antibodies. To detect the expression of TF and PS on ECs, treated ECs were stained with anti-CD142 antibody (APC, Biolegend, 365205) with FITC-conjugated bovine lactadherin (Hematologic Technologies). The flow cytometry strategies are shown in Supplemental Figure 1. All samples were fixed with 1% paraformaldehyde prior to analysis by flow cytometry (BD, FACSCanto II).

### ELISA.

The levels of TAT complex, VWF, syndecan-1, and thrombomodulin in samples were detected by Human TAT complex ELISA kit, Human VWF ELISA kit, Human Syndecan-1 ELISA kit, and Human Thrombomodulin ELISA kit (all from Jingkangbio). For detecting D-dimer and fibrinogen in mouse plasma, a Mouse D-dimer ELISA kit and Mouse Fibrinogen ELISA kit (both from Jingkangbio) were utilized according to the manufacturer’s protocol. For detecting citH3-DNA in plasma from mice, we used a modified capture ELISA method. We first used a Mouse citH3 ELISA kit (Jingkangbio) to capture citH3 in plasma from each group. Then, the Quant-iT PicoGreen dsDNA assay kit (Invitrogen) was used according to the manufacturer’s instructions to determine DNA concentration, defined as citH3-DNA concentration (μg DNA/mL).

### Statistics.

The Shapiro-Wilk test was used to test normal distribution of the data sets. To compare 2 groups, Wilcoxon’s test and unpaired 2-tailed *t* test were used for paired samples and Mann-Whitney and unpaired *t* test with Welch’s correction were used for unpaired samples as needed. For comparisons among more than 2 groups, ordinary 1-way ANOVA with Turkey’s multiple-comparison test, Brown-Forsythe and Welch’s ANOVA test, Kruskal-Wallis with Dunn’s multiple-comparison test, and repeated measures ANOVA were used as needed. All analyses were performed with GraphPad Prism 9.0. A *P* value of less than 0.05 was considered statistically significant.

### Study approved.

Human experimental protocols were approved by the local ethics committee of the Second Affiliated Hospital of Harbin Medical University. For collecting human samples, written informed consent was obtained before screening. The mouse studies were approved by the Institutional Animal Ethics Committee of the Harbin the Second Affiliated Hospital of Harbin Medical University.

## Author contributions

JJ and FW designed the study, performed experiments, analyzed the results, made the figures, wrote the manuscript, analyzed data, and revised the manuscript. JT, XZ, JD, NW, ZL, and HZ performed some experiments. WL, GM, and SH obtained funding, designed the study, performed experiments, analyzed the results, made the figures, and revised the manuscript.

## Supplementary Material

Supplemental data

## Figures and Tables

**Table 1:**
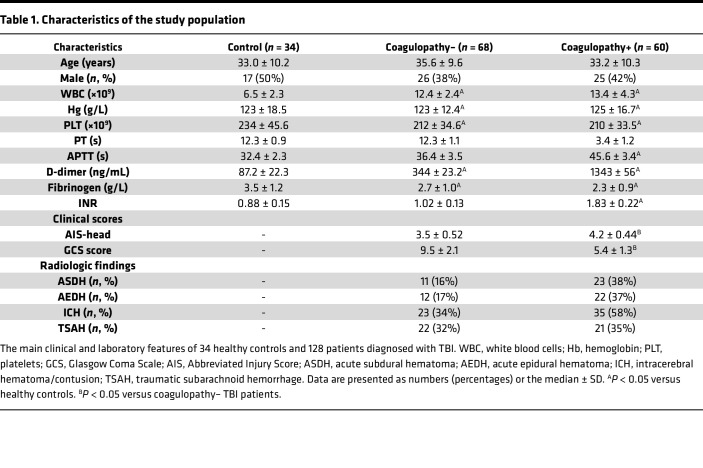
Characteristics of the study population

## Version 1 02/21/2023

In-Press Preview

## Version 2 03/22/2023

Electronic publication
