# Supplementary material for: Neutrophil extracellular traps contribute to coagulopathy after traumatic brain injury
Source: JCI Insight. 2023 Mar 22;8(6):e141110. doi: 10.1172/jci.insight.141110 (PMC10070118; doi:10.1172/jci.insight.141110)
Supplement: Supplemental data [file jciinsight-8-141110-s235.pdf]

Supplementary Materials

NETs isolation

For NETs isolation (Figure 3I-L, Figure 4D, E, G-I, Figure 5, Supplementary Figure 3 and 6), isolated neutrophils (5 x 10^6 neutropils/ml) from healthy subjects were incubated with 500 nM PMA for 4 h. To lift off all adherent NETs from the bottom, we removal of the supernatant and wash the bottom of each dish by pipetting 15 ml of cold PBS on the bottom of the dish and collected solution, centrifuge for 10 min at 450 x g at 4 °C. Then, we divided supernatant into 1.5 ml micro-centrifuge tubes and centrifuge for 10 min at 18,000 x g at 4 °C.

For NETs isolation (Figure 4B, 4C, supplemental Figure 8), 1.5x10^6 purified neutrophils from healthy subjects (Control NETs) and TBI patients (coagulopathic patients NETs and non-coagulopathic patients NETs) were seeded in six-well culture plates in RPMI culture medium for 4h (37°C and 5% CO_2)(1, 2). Following incubation, the culture medium was removed, and each well was washed twice with pre-warmed RPMI. To isolate in vitro generated NET structures, 750ul of fresh RPMI were added in each well and NETs adherent to the plate were collected after vigorous agitation. The medium was centrifuged at 20x g for 5 min and supernatant phase, containing NETs, was collected and stored at -20°C until use. Measure DNA concentration in isolated NETs (μgDNA/ml) using spectrophotometry and we stored isolated NETs at -80°C for further experiment.

NETs stimulation

Human neutrophils were isolated from healthy subjects or patients by using human neutrophil separation medium (TBD, Tianjin) according to the manufacturer's protocol. Purity of neutrophils was >95% (Wright–Giemsa staining). To compare the ability of NETs generation, neutrophils from each group were cultured in 5% CO2 at 37°C in RPMI (Gibco) for 4 h (Figure 2B-E). To investigate the role of platelets on NETosis, control neutrophils were incubated with PRP (20%) or platelets(plateltes:neutrophils=20:1) from citrate blood of healthy subjects and TBI patients for 2 hours . To determine if HMGB1 induced NET formation through autophagy, recombinant HMGB1 (HMG Biotech, 10 μg/mL), Box A

(HMG Biotech, 10 μg/mL), HCQ (MedChemExpress, HY-B1370, 50μM,) were incubated with neutrophils (1 x 10^5 cells per well) for 1 hour. To detect the interaction between NETs and coagulation factors, isolated NETs we incubated with DNase I (100 U/mL, Merck), lactadherin (200 nM, Novus), anti-TF antibody (40 μg/ml) and anti-IgG antibody for 1 h and cultured with platelet-poor plasma for 10 min at 37°C.

NETs quantification

For NETs quantification, we detected NET–DNA complex (MPO-DNA, NE-DNA, citH3-DNA, PS-DNA, TF-DNA, fibrinogen-DNA, prothrombin-DNA, factor X-DNA) using a modificated capture ELISA method. We first used myeloperoxidase (MPO) ELISA kit (Jingkangbio, Shanghai), neutrophil elastase (NE) ELISA kit (Jingkangbio, Shanghai) and citH3 ELISA kit (Jingkangbio, Shanghai), human PS ELISA kit (Jianglaibio, Shanghai), human TF ELISA kit (Cloud Clone), fibrinogen ELISA kit (Jingkangbio, Shanghai), prothrombin ELISA kit (Jingkangbio, Shanghai), factor X ELISA kit (Jingkangbio, Shanghai) to capture MPO, NE, citH3, PS, TF, fibrinogen, prothrombin, factor X in plasma respectively, according to the manufacturer's protocol. Then, Quant-iT PicoGreen dsDNA assay kit (Invitrogen) was added to per well according to manufacturer's directions to detect DNA concentrations, defined as NET-DNA concentrations. Cell-free DNA (Cf-DNA) was also detected by Quant-iT PicoGreen dsDNA assay kit (Invitrogen) according to manufacturer's directions.

Coagulation assays

To dectect the generation of intrinsic FXa, platelets (1 x 10^5) were incubated with DNase I (100 U/mL, Biolabs), activated protein C (APC, 100 nM, Med Chem Express), and sivelesatat (100 nM, Med Chem Express). Then treated platelets were incubated with a mixture of coagulation factor (1 nM factor IXa, 130nM factor X, 0.2 nM thrombin and 5 nM factor VIII) and $CaCl_2$ (5 mM) in factor Xa buffer (1 ml 10xTBS, 200 μl 10% BSA and 8.8 ml ddH2O) for a final reaction volume of 40 μl. After 5 min at 25 °C, the reaction was stopped by the

addition of EDTA (7 mM, final concentration). FXa generation was estimated immediately with the chromogenic substrate S-2765 (0.8 mM). Optical densities at 405 nm were recorded to represent the rate of substrate cleavage of a standard dilution of FXa. The extrinsic activation of factor X by factor VIIa was also measured in factor Xa buffer. The platelets (1 x 10^5) were mixed with 130 nM factor X, 1 nM factor VIIa and 5 mM Ca^{2+} instead. The amount of FXa formed was determined as described above. Prothrombinase formation was measured as follows: platelets (1 x 10^5) were incubated with 1 nM FVa, 0.05 nM FXa, 1 μM prothrombin and 5 mM Ca^{2+} in prothrombinase buffer (1 ml 10 x TBS, 50 μl 10% BSA and 8.95 ml ddH2O) for 5 min at 25 °C. Thrombin production was assessed after the addition of EDTA and chromogenic substrate S-2238, as mentioned above. Human alpha-thrombin (IIa) human factor X, Xa, IXa, VIIa, VIII and thrombin were obtained from Enzyme Research Laboratories (South Bend, IN). Chromogenic substrates S-2765 and S-2238 were obtained from Instrumentation Laboratory Company (MA, USA).

Supplement Figure 1

(A) Flow cytometry analysis of NETing neutrophils from whole blood. (B) The flow cytometry strategy of platelet-neutrophil aggregates. (C) The flow cytometry strategy of platelets in whole blood.

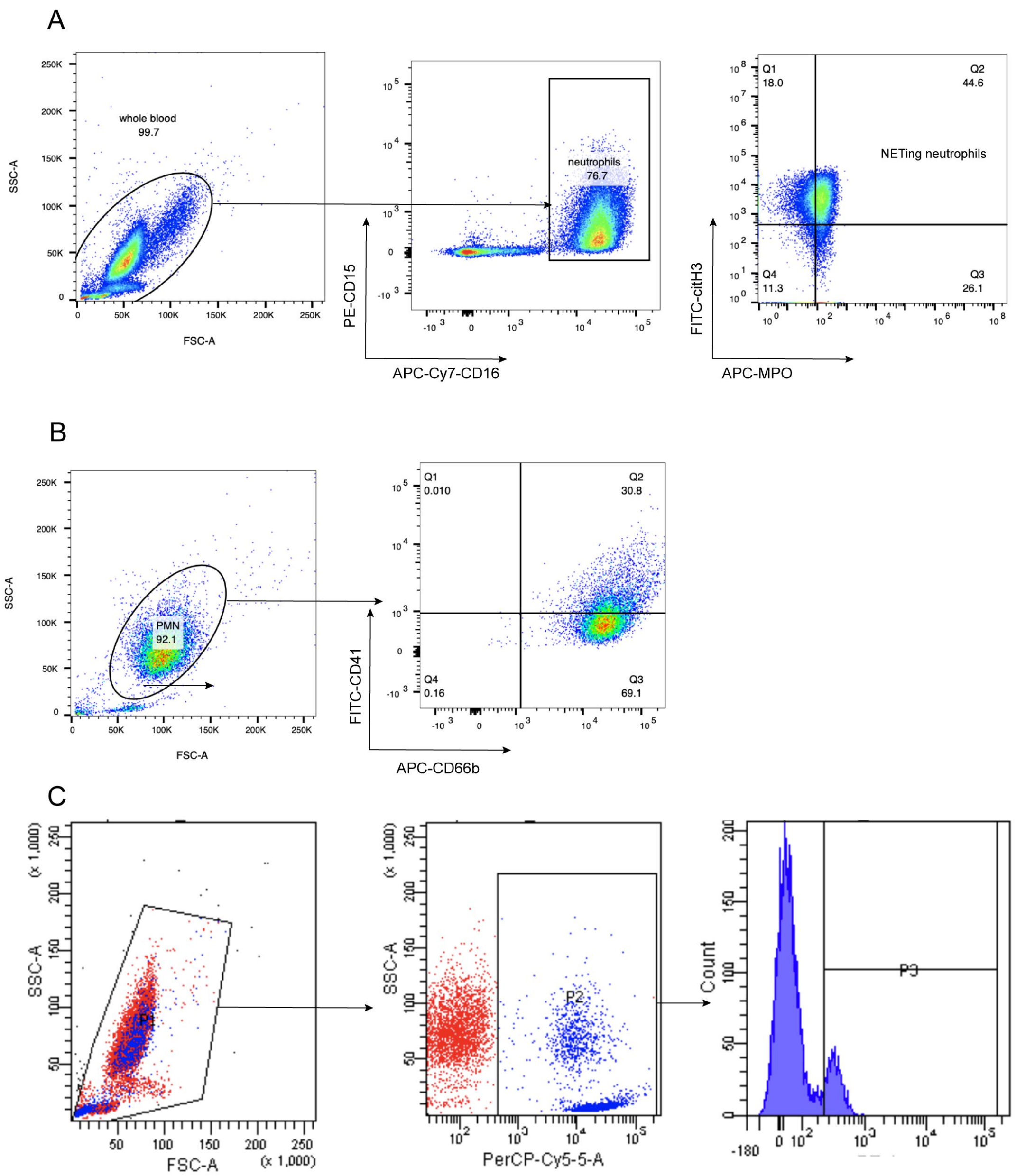

Supplement Figure 2

Control neutrophils were incubated with recombinant HMGB1 protein at different concentrations. The concentrations of PS-DNA (A) and TF-DNA (B) in the culture supernatant from each group. (C-F) Representative confocal images of PS (green) and TF (red) on neutrophils incubated with HMGB1. The inset scale bar in C-F is 20 μm. One representative out of five (H and J) independent experiments is shown. Statistics, one-way ANOVA with Tukey's multiple comparisons test. Data are presented as the mean ± SD. ****P< 0.0001.

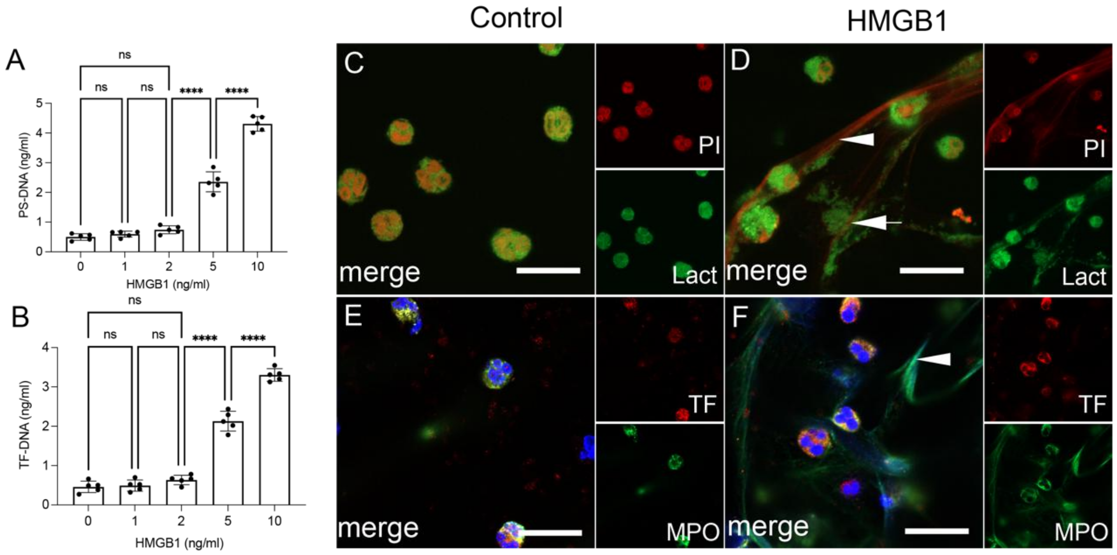

Supplement Figure 3

The expression of HMGB1 on isolated NETs, control PLTs treated with or without NETs.

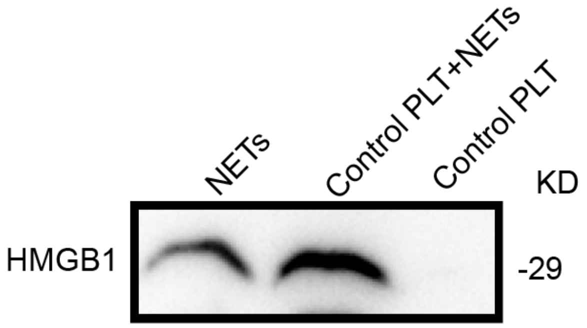

Supplement Figure 4

(A-C) The TAT complex was positively correlated with the NET markers citH3-DNA, MPO-DNA, and NE-DNA in plasma samples from TBI patients with coagulopathy. Statistics, Spearman correlation test(A-C).

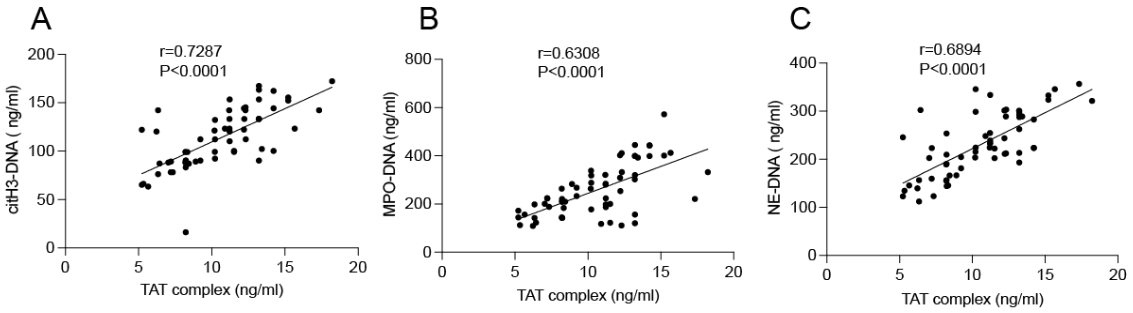

Supplement Figure 5

NETing neutrophils were incubated with plasma and stained with coagulation factors, including fibrinogen (red), prothrombin (red), factor X (red) and MPO (green). (A-C) Representative confocal images of colocalization of NETs and coagulation factors. The inset scale bar in A-C is 20 μm.

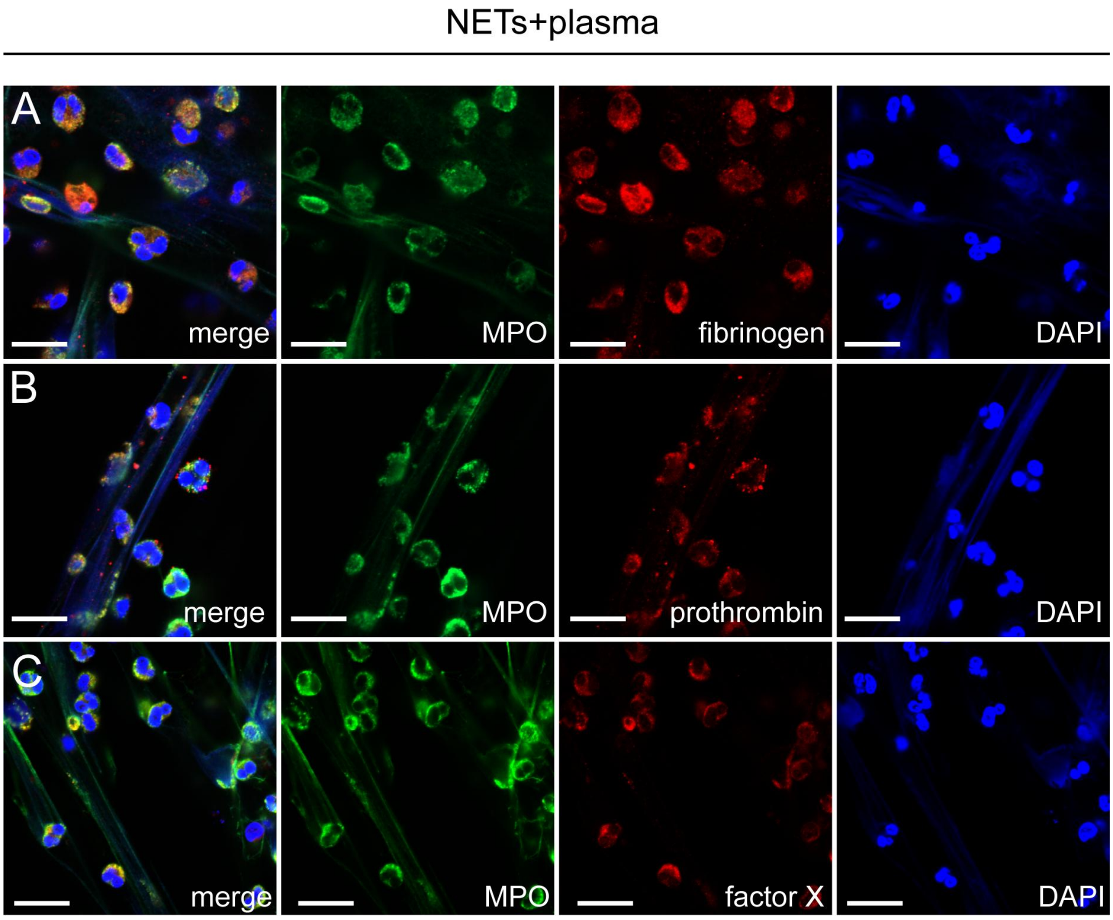

Supplement Figure 6

Control platelets were incubated with isolated NETs. intrinsic Xa (A), extrinsic Xa (B) and thrombin (C) production in platelets were dependent on NETs concentrations, and these effects could be significantly diminished by NETs inhibitors. (D) Representative confocal images of fibrin formation in platelets. The inset scale bar in D is 20 µm. One representative out of eight (A-C) independent experiments is shown. Statistics, Kruskal-Wallis test with Dunn's multiple comparisons test(A-C). Data are presented as the mean ± SD. **P< 0.01 and ****P< 0.0001.

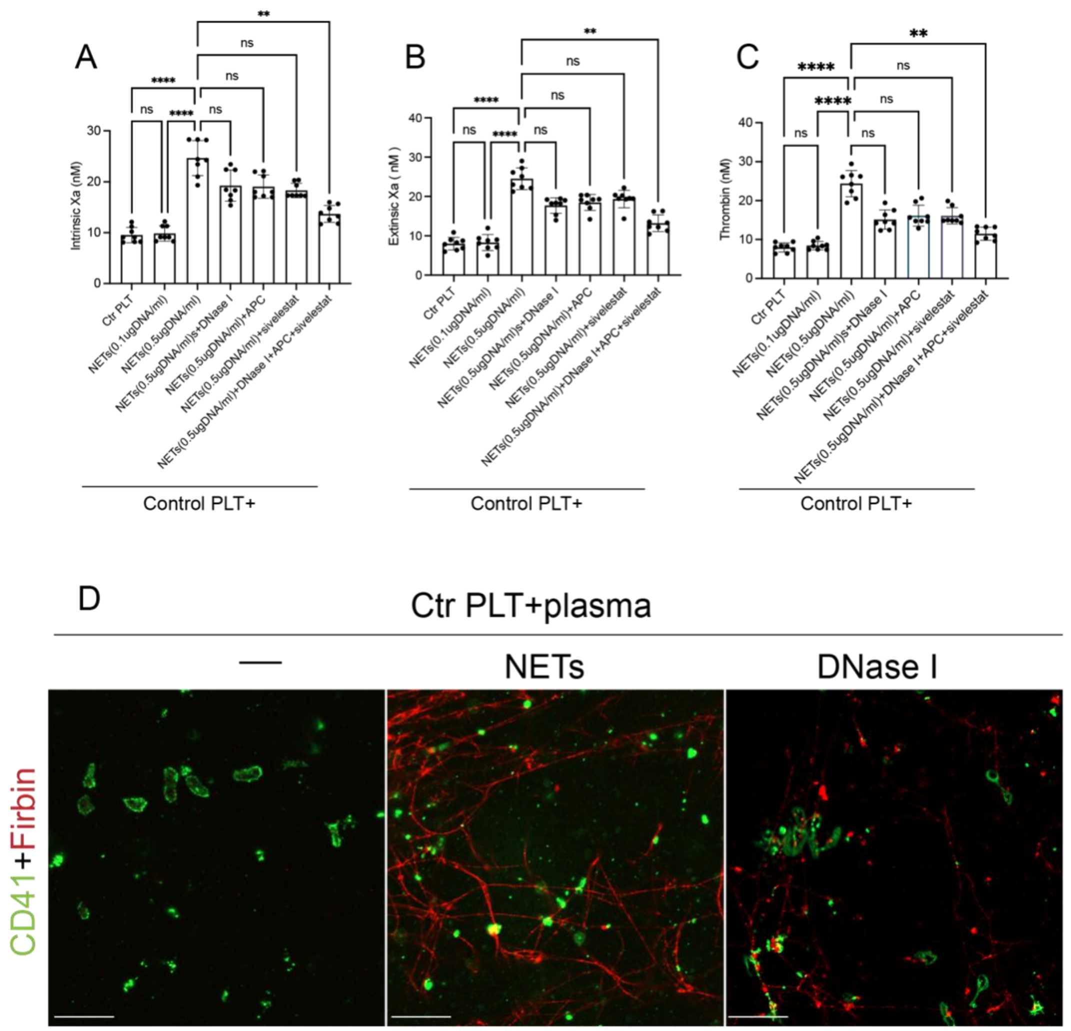

Supplement Figure 7

The plasma levels of the endothelial markers syndecan-1 (A), soluble thrombomodulin (B) and VWF(C) were detected in samples from each group by ELISA. These markers were significantly elevated in plasma from coagulopathic patients compared with other groups. Syndecan-1 (D), soluble thrombomodulin (E) and VWF(F) were positively correlated with citH3-DNA in samples from coagulopathic patients. Statistics, Kruskal-Wallis test with Dunn’s multiple comparisons test(A-C), Spearman correlation test(D-F). Data are presented as the mean ± SD. *P < 0.05, **P < 0.01, ***P< 0.001 and****P< 0.0001.

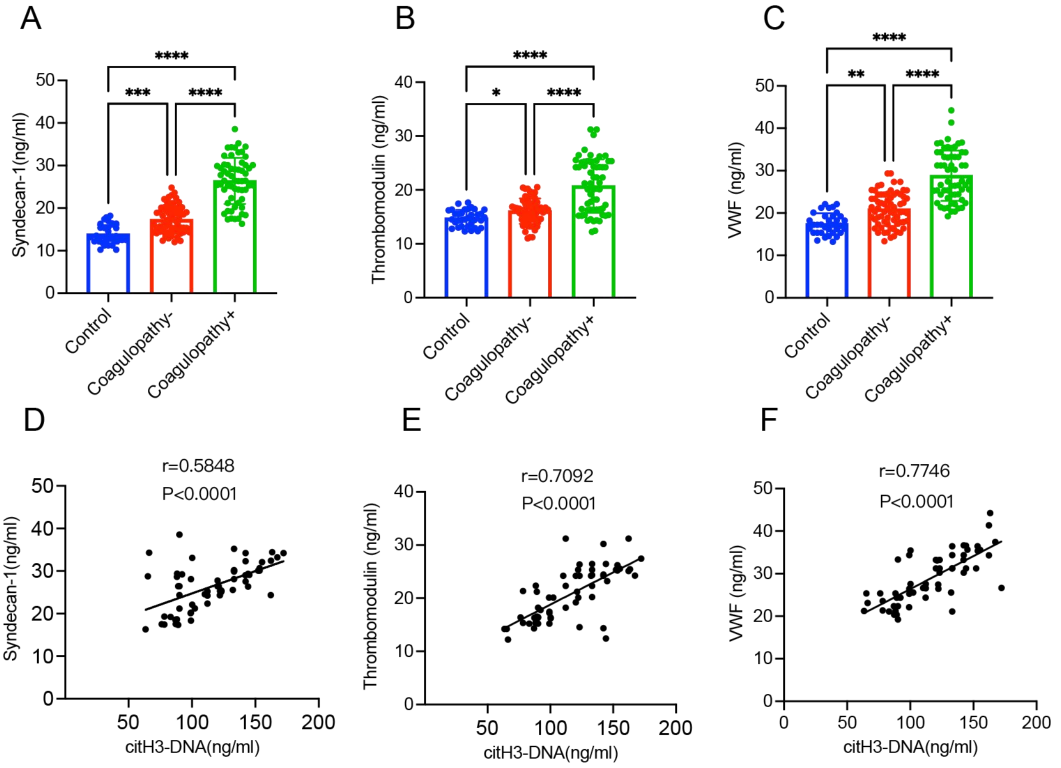

Supplement Figure 8

ECs were incubated with NETs from healthy subjects and TBI patients and stained with syndecan-1, soluble thrombomodulin and VWF. ELISA results suggested that syndecan-1 (A), soluble thrombomodulin (B) and VWF(C) levels in the supernatant of treated ECs. Statistics, Kruskal-Wallis test with Dunn's multiple comparisons test(A-C), Data are presented as the mean ± SD. *P< 0.05, **P< 0.01 and ****P< 0.0001.

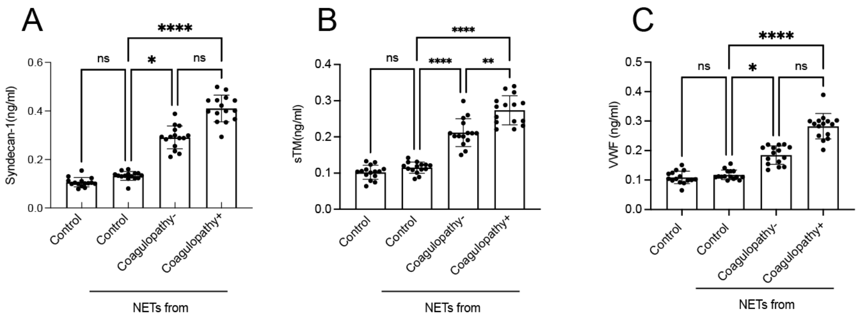

Supplement Figure 9

Represented confocal microscopy images of brain tissues from sham and TBI mice. NETs were stained with citH3(green) and Ly6G(red). The inset bar is equal 40 µm.

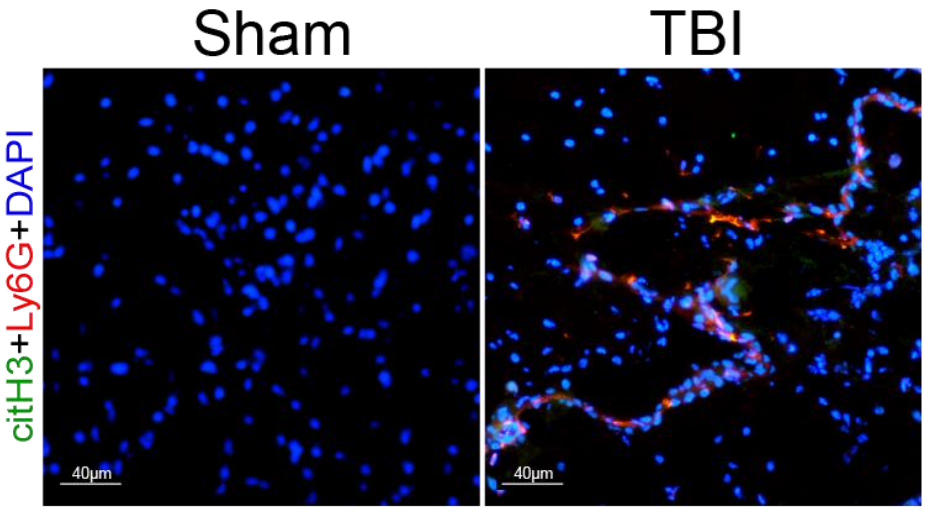

Supplement Figure 10

Phosphotungstic acid hematoxylin stainingof the lungs(A-C) and kidneys(D-F) from sham, TBI+Saline and TBI+DNase I. Phosphotungstic acid hematoxylin staining indicated widespread fibrin deposition (black, arrowbar) in the microvasculature of the lungs and kidney of TBI mice but not in the same organs of TBI mice treated with DNase I. The inset bar in A-F is equal 40 μm.

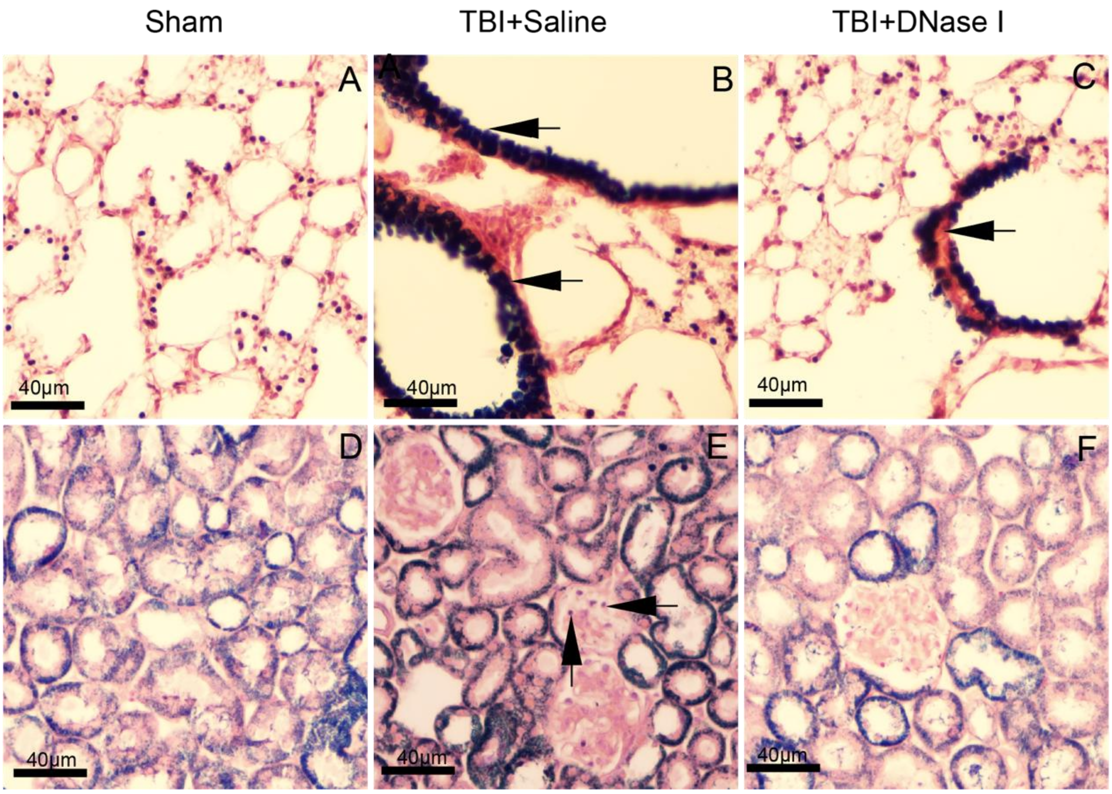

References

1. Chrysanthopoulou A, Gkaliagkousi E, Lazaridis A, Arelaki S, Pateinakis P, Ntinopoulou M, et al. Angiotensin II triggers release of neutrophil extracellular traps, linking thromboinflammation with essential hypertension. *JCI Insight.* 2021;6(18).
2. Stakos DA, Kambas K, Konstantinidis T, Mitroulis I, Apostolidou E, Arelaki S, et al. Expression of functional tissue factor by neutrophil extracellular traps in culprit artery of acute myocardial infarction. *Eur Heart J.* 2015;36(22):1405-14.
